# Human judgement forecasting tournaments: A feasibility study based on the COVID-19 pandemic with public health practitioners in England^☆^

**DOI:** 10.1016/j.puhip.2022.100260

**Published:** 2022-04-22

**Authors:** Nathan Davies, Simon Ferris

**Affiliations:** a University of Nottingham, School of Medicine. Clinical Sciences Building, Nottingham City Hospital, Hucknall Road, Nottingham, NG5 1PB, UK; b University of Nottingham, School of Medicine, Nottingham, UK

**Keywords:** Forecasting, Public health policy, Public health training, Tournaments, COVID-19

## Abstract

**Objectives:**

To explore the viability of running human judgement forecasting tournaments with public health practitioners, and to gather initial data on forecasting accuracy and participant perceptions of forecasting.

**Study design:**

Quality improvement study comprising two COVID-19 forecasting tournaments using Brier Skill Score scoring and a follow-up participant questionnaire.

**Methods:**

Over two forecasting tournaments, public health registrars in the East Midlands, UK, assigned probabilities to future possible binary events relating to COVID-19. Participants also completed a questionnaire on their experiences of forecasting.

**Results:**

There were 17 participants in the first tournament and nine in the second tournament, with no new participants. In both tournaments, the majority of participants scored a Brier Skill Score above the benchmark of 0. The median Brier Skill Score improved slightly between the two tournaments. Participants reported luck and changing political climates as impacting their performance. Participants reported forecasting in their day job but had received no formal training to do so.

**Conclusions:**

Forecasting is an important public health skill, and human judgement forecasting tournaments can be run amongst public health practitioners with little time and resource requirements. Further research would help identify whether training, teamwork or other interventions can improve public health forecasting accuracy.

## 1 Introduction

Epidemiological forecasting has had unprecedented coverage during the COVID-19 pandemic. National models such as those developed by Imperial College London, University of Washington Institute for Health Metrics and Evaluation and the University of Warwick have informed policymaking and public debate [1].

Much less attention has been given to the informal forecasts made by local and regional public health practitioners every day. Whereas statistical models have been developed to support local decision-making [2] there is far less evidence on how forecasts on questions that require a large degree of human judgement are made, presented and tracked.

Throughout the pandemic, practitioners have been asked by policymakers to speculate on the short, medium, and long-term policy decisions and disease-related outcomes for their local area. Whereas local practitioners can draw upon formal national and local models of disease burden, forecasts on the national policy landscape and local project trajectories will likely be based on several, more informal, sources of information. Beyond COVID-19, public health practitioners will use judgement to make forecasts in day-to-day work, such as the content of a new national obesity strategy, the length of time to complete a particular project, and the allocation for their departmental budget next financial year.

The broader field of improving human judgmental forecasting, and integrating human judgment into quantitative forecasts, is well-developed [3]. One simple way of tracking forecast performance is through forecasting tournaments. Tournaments enable precise recording, tracking and evaluating the forecasts of many individuals or teams on the same questions [4]. Failing to track accuracy of forecasts may contribute to poor performance and missed opportunities to learning from mistakes [5]. Tournaments have been used to develop and evaluate training to improve forecasting performance and to identify those with higher skill in forecasting, popularised as “superforecasters” [4].

It is possible that public health practitioners making, tracking, and scoring quantified predictions could lead to better quality, more reflective forecasting. This study created a safe environment for public health practitioners to participate in a forecasting tournament, and to learn from each other's successes and failures.

## 2 Methods

Our tournament participants were public health registrars based in the East Midlands, who were at the time on a wide range of placements with different agencies with public health responsibilities. Ethical approval was not required as this was a quality improvement project focused on improving the judgement of public health professionals.

Participants were invited to join two tournaments through the East Midlands (UK) public health registrar emailing list (n = 17). Participants were given a brief information sheet explaining the forecasting process and scoring system. They were asked to assign a probability between 0 and 1 to several epidemiological or policy-related events taking place by a given date, where 0 = certain non-occurrence, 0.5 = equal likelihood and 1 = certain occurrence. Participants could not change their forecast after set cut-off dates following tournament launches. All forecasts were for events up to three months into the future.

There were nine questions for the first tournament and eight in the second (see Supplementary Materials 1 for question sets). The tournaments used cumulative Brier scores (the square deviation between the forecast and the outcome, scored as 0 for non-occurrence and 1 for occurrence) [6]. Results were summed to give each participant a total Brier score and a comparative score. For this analysis, a Brier skill score (BSS) was calculated for each participant for each tournament. A participant would receive a BSS of 0 for assigning 0.5 probability to each event, a BSS of 3 for correct predictions made with certain confidence and a BSS of -1 for incorrect predictions made with certain confidence.

The first tournament ran from June 19, 2020 to September 6, 2020. The second tournament ran from October 23, 2020 to November 16, 2020.

A follow-up questionnaire was sent out in November 2021 to participants to ask about their experiences with forecasting and of the tournament.

## 3 Results

There were 17 participants in the first tournament and nine in the second tournament, with no new participants.

**Fig. 1:**
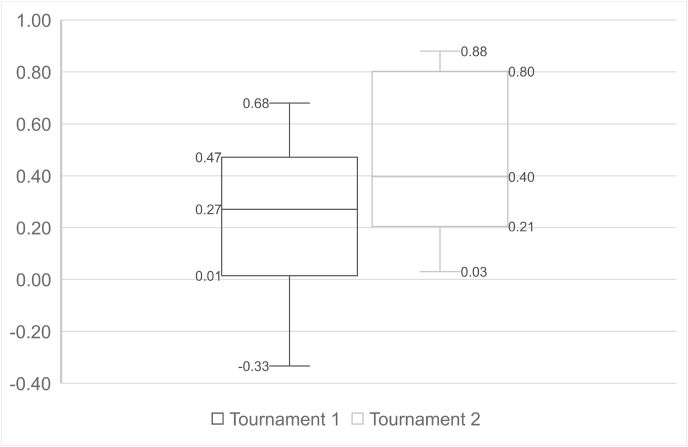
Box and whisker plot of Brier skill scores across tournaments.

The best-performing participant scored a BSS of 0.88, with the lowest score being -0.33. In both tournaments the majority of participants performed better than the benchmark (a rational person with no information, who would score 0). The median BSS was 0.27 in the first tournament and 0.40 in the second tournament, with no participant receiving a negative BSS in the second competition. Fig. 1 shows a box and whisker plot of the Brier skill scores for participants for tournament 1 and tournament 2.

The BSS range between participants decreased from the first to second tournament from 1.01 to 0.85, although the interquartile range slightly increased from 0.35 to 0.48. The lowest end of the range in Tournament 2 was 0.03, meaning all participants scored higher than the benchmark of 0.

Those who participated in both tournaments scored similarly to those who participated in the first tournament alone, with an average BSS of 0.19 compared to the first tournament average of 0.23.

Those who participated in both tournaments saw a mean point difference in BSS of 0.27, although 3 out of 9 performed better in the first tournament. There was minimal correlation between performance in the first and second tournament for participants, with a Spearman's rank correlation coefficient for those who performed in both rounds of 0.3. The mean length of time spent making predictions was 7.5 min.

Eight participants responded to the follow-up questionnaire. Seven respondents had been involved in making forecasts whilst in role as a public health registrar. None reported receiving training in forecasting or prior experience of using Brier scores for making predictions. One participant reported changing their strategies between tournaments, to make predictions with higher confidence.

Some participants reported difficulties making predictions, citing political unpredictability and luck as factors in accuracy. One commented that forecasting accuracy may be improved in a less volatile environment. Participants did not express being markedly more confident in making predictions after these competitions. Participants reported forecasting was an enjoyable and interesting experience, although one expressed concern at the use of “tournaments” relating to COVID-19 prediction.

## 4 Discussion

This paper shows that simple forecasting tournaments can be implemented at local or regional level with very little time and resource, with participants taking 7.5 min on average to make predictions. We found that participants demonstrated some skill in predicting outcomes, which does not hold for all expert groups [5], and that performance improved between tournaments. Possible reasons for improvement between tournaments include participants improving their forecasting, participants having a better understanding of the Brier scoring method, questions in the second tournament being easier to predict, or participants who stayed on for Tournament 2 having greater skill or interest in forecasting.

The chief limitations of this quality improvement project include the small sample size of both participants and questions. There was also a gap between the tournaments and administering the participant questionnaire, which could have led to recall bias given the evolving pandemic knowledge.

However, this study suggests more could be done to improve the monitoring and accuracy of the predictions public health professionals make. For example, nearly all questionnaire respondents reported making forecasts in the course of their public health work, though none reported past training in forecasting, and only one reported changing strategy. The wider forecasting literature indicates that training, constantly adapting one's approach, making predictions in teams and using probabilistic thinking can improve predictive accuracy [4]. This could be tested in public health through future research which randomises participants to differing interventions mid-tournament. This is important because accurate forecasts can support good quality public health work. For example, accurately predicting that a government public health strategy will be delayed by more than a year may lead a local area to take earlier action themselves rather than wait for central directives.

## 5 Conclusion

Human judgement forecasting is a de facto part of public health practice, although it is rarely explicitly recognised as such. A key component of prevention is the ability to predict, and public health registrars are expected to make forecasts and predictions as part of their work on placements. However, participants stated low levels of confidence in forecasting methods and strategy choice. They also reported a lack of formal training.

Public health professionals may benefit from training in human judgement forecasting to improve their future predictions, which should be explored by further research. Better forecasting skills and an understanding of how to use past forecasts to refine future predictions may have beneficial real-world effects, leading to better reflective public health practice, and ultimately improvements in public health.

## Declaration of competing interest

The authors declare that they have no known competing financial interests or personal relationships that could have appeared to influence the work reported in this paper.
