# Supplementary material for: Human judgement forecasting tournaments: A feasibility study based on the COVID-19 pandemic with public health practitioners in England
Source: Public Health Pract (Oxf). 2022 Apr 22;3:100260. doi: 10.1016/j.puhip.2022.100260 (PMC9023363; doi:10.1016/j.puhip.2022.100260)
Supplement: Multimedia component 1 [file mmc1.docx]

**Forecasting tournament questions: Tournament 1**

1. All primary school aged children in England will be able to return to school full-time for the first school days in September if their parents wish.

2. All secondary school aged children (11-18) in England will be able to return to school full-time for the first school days in September if they/their parents wish.

3. Local authorities in England will be given new 'local lockdown' powers by 1st August.

4a. The 7-day average number of daily confirmed cases in the UK will be below 1,000 for the week starting 29th June (when viewed on https://coronavirus.data.gov.uk/ on 12th July to allow for late reporting)

4b. The 7-day average number of confirmed cases in the UK will be below 500 for the week starting 29th June (when viewed on https://coronavirus.data.gov.uk/ on 12th July)

4c. The 7-day average number of daily confirmed cases in the UK will be below 100 for the week starting 29th June (when viewed on https://coronavirus.data.gov.uk/ on 12th July)

5. The weekly number of total deaths for the UK reported by ONS will be below the 5-year average for UK, for the week ending 12th June (see figure 1 at https://www.ons.gov.uk/peoplepopulationandcommunity/birthsdeathsandmarriages/deaths/bulletins/deathsregisteredweeklyinenglandandwalesprovisional/weekending22may2020#deaths-registered-by-week)

6. There will be no guidance in England on visiting other people's private home by 1st September 2020 (notwithstanding shielding advice).

7. The two-metre social distancing rule will be cut to one metre or less, or removed, in England by 1st July 2020.

How long, in minutes and hours, did you spend on your predictions?

**Forecasting tournament questions: Tournament 2**

1. England and Wales will have statistically significant excess deaths for the week ending 30th October (see point 2 of https://www.ons.gov.uk/peoplepopulationandcommunity/birthsdeathsandmarriages/deaths/bulletins/deathsregisteredweeklyinenglandandwalesprovisional/weekending25september2020)

2. Nottingham City will be moved up to "very high" tier of national restrictions by 8th November 2020.

3. Derby City will be moved up to the "very high" tier of national restrictions by 8th November 2020.

4. Lincoln City will be moved to the "very high" tier of national restrictions by 8th November 2020.

5. One or more local authority area who are in the "high" tier of national restrictions on 14th October 2020 are moved down to the "medium" tier of restrictions by 8th November 2020.

6. The average number of deaths per day for 2nd November - 8th November will be greater than 100, as given by coronavirus.data.gov.uk on 16th November

7. Sixth form colleges and/or universities will be physically closed in parts of the UK following government advice on 16th November 2020.

8. An English CMO or deputy CMO will announce their resignation by 16th November 2020.

How long, in minutes and hours, did you spend on your predictions?
